# Saliva and Plasma Neutralizing Activity Induced by the Administration of a Third bnt162b2 Vaccine Dose

**DOI:** 10.3390/ijms232214341

**Published:** 2022-11-18

**Authors:** Micaela Garziano, Olga Utyro, Sergio Strizzi, Claudia Vanetti, Irma Saulle, Chiara Conforti, Federica Cicilano, Francesco Ardizzone, Gioia Cappelletti, Mario Clerici, Fiona Limanaqi, Mara Biasin

**Affiliations:** 1 Department of Biomedical and Clinical Sciences, University of Milan, Via G.B. Grassi, 20122 Milan, Italy; 2 Department of Pathophysiology and Transplantation, University of Milan, Via Francesco Sforza, 20122 Milan, Italy; 3 Don C. Gnocchi Foundation, Istituto di Ricovero e Cura a Carattere Scientifico (IRCCS) Foundation, Via A. Capecelatro 66, 20148 Milan, Italy

**Keywords:** SARS-CoV-2, saliva, neutralizing activity, vaccine, variants, booster dose

## Abstract

The BNT162b2 vaccine induces neutralizing activity (NA) in serum, but no data are available on whether a third-dose activates specific-immunity within the oral mucosa, representing the primary route of viral-entry. To carefully address this issue, we investigated if such immunity is boosted by SARS-CoV-2-infection; how long it is maintained over-time; and if it protects against the SARS-CoV-2 lineage B.1 (EU) and the emerging Delta and Omicron variants. NA was measured in plasma and saliva samples from: uninfected SARS-CoV-2-Vaccinated (SV), subjects infected prior to vaccination (SIV), and subjects who were infected after the second (SIV2) or the third (SIV3) vaccine dose. Samples were collected immediately before (T0), 15 days (T1), and 90 days (T2) post third-dose administration (SV and SIV), or 15 days post-infection (SIV2 and SIV3). In all the enrolled groups, NA in plasma and saliva: (i) was higher against EU compared to the other variants at all time-points (SV: T0 and T1, EU vs. both Delta and Omicron *p* < 0.001; T2 *p* < 0.01) (SIV: T0, EU vs. Delta *p* < 0.05; EU vs. Omi *p* < 0.01; T1 and T2 EU vs. Delta *p* < 0.01; EU vs. Omi *p* < 0.001); (ii) was boosted by the administration of the third dose; iii) declined over-time, albeit being detectable in almost all subjects at T2. The monitoring of NA over time will be important in clarifying if different NA levels may influence either acquisition or course of infection to properly plan the timing of a fourth vaccine dose administration.

## 1. Introduction

Mucosal immunity is the body’s first line of defence against respiratory viruses, including the novel coronavirus SARS-CoV-2 (severe acute respiratory syndrome coronavirus 2), the causative agent of the Coronavirus Disease 19 (COVID-19). In fact, besides the epithelia of the lung, and small intestine among others [1], the main SARS-CoV-2-receptor angiotensin-converting enzyme 2 (ACE2), is expressed in the mucosa of the upper respiratory tract [2], making it highly susceptible to SARS-CoV-2 infection [3]. Yet, the prompt activation of several non-specific antimicrobial mediators, such as agglutinins, mucins, macrophages, dendritic cells, and innate lymphoid cells, coupled with the tightly composed endothelial cells, enable the oral epithelium to provide protection in the very first phase of infection [4,5,6,7]. This shield is further strengthened by the salivary secretion of proinflammatory cytokines and chemokines, which in turn elicit antigen-specific T and B lymphocyte responses [8]. All of these events contribute to generating the so-called neutralizing activity (NA), which is mainly driven by neutralizing antibodies and, in most cases, can efficiently control the spreading of infection.

The introduction of the COVID-19 vaccines has substantially reduced the risk of clinical fatality [9,10,11]. In particular, the administration of two mRNA-based vaccine-doses (BNT162b2, Pfizer/BioNTech) has proved to trigger a robust NA at the systemic level [12], thus preventing the onset of severe COVID-19, meanwhile providing some degree of protection against infection/reinfection [13,14]. This observation suggests that, despite being administered systemically, the BNT162b2 vaccine is able to elicit defensive immunity locally within the mucosal site, which may contribute to overall protection against viral infection. Therefore, assessing the NA in oral mucosa might provide key information about vaccine efficacy in safeguarding the host from the infection, while contributing to properly scheduled vaccination campaigns.

However, due to anti-SARS-CoV-2 NA decay over time [15], a third vaccine booster dose was introduced in many countries; several studies are ongoing to verify if it is able to quickly evoke and re-activate immunity and produce sufficient and durable immune protection at both systemic and local levels. This requirement is far more urgent when considering the impact of antigenic viral variants of concern (VOCs) that have recently emerged with spike protein mutations, presumably as a consequence of suboptimal immunity in areas of high virus spreading [16]. In fact, these VOCs have been shown to somewhat elude neutralization by monoclonal antibodies, convalescent serum samples, and vaccine-elicited serum samples [17,18,19,20], and inadequate neutralization may hinder vaccine efficacy.

Based on these premises, herein we investigated the immune response to a third booster dose of SARS-CoV-2 BNT162b2 vaccination in 122 volunteers by measuring their NA [21] and cytokine/chemokine release in both saliva and plasma samples collected from November 2021 to May 2022 at regular intervals over a 3-month period. Given the potential for antigenic variation to lead to vaccine breakthrough, NA was tested against SARS-CoV-2 lineage B.1 (EU) and two alarming VOCs: B.1.617.2 (Delta) and B.1.1.529 (Omicron) (https://www.who.int/en/activities/tracking-SARS-CoV-2-variants/ accessed date 28 October 2022). Finally, to determine the extent to which prior infection influences the response to vaccination, results were compared to those of subjects who were infected by SARS-CoV-2 in different pandemic waves.

## 2. Results

### 2.1. SARS-CoV-2 Neutralizing Activity (NA) in Plasma

**Figure 1:**
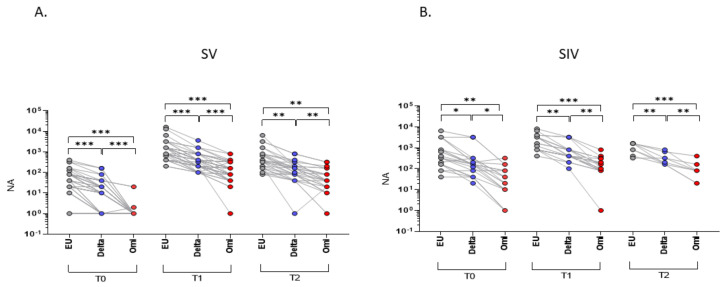
Plasma neutralizing activity (NA) correlations among different VOCs. NA correlation performed at different time points (T0, T1, and T2) showed a higher activity against the EU variant compared to the Delta and Omicron (Omi) ones in plasma samples from both SARS-CoV-2 Vaccinated subjects (SV) (**A**) and SARS-CoV-2 Infected and Vaccinated subjects (SIV) (**B**). Lines connect the NA of each subject. * *p* < 0.05, ** *p* < 0.01, *** *p* < 0.001.

As expected, NA against the SARS-CoV-2 EU strain was significantly higher compared to both the Delta and Omicron strains at all-time points in both SV (T0 and T1, EU vs. Delta and EU vs. Omicron: *p* < 0.001; T2, EU vs. Delta and EU vs. Omicron: *p* < 0.01) and SIV (T0: EU vs. Delta: *p* < 0.05; EU vs. Omicron: *p* < 0.01; T1: EU vs. Delta: *p* < 0.01; EU vs. Omicron: *p* < 0.001; T2: EU vs. Delta: *p* < 0.01; EU vs. Omicron: *p* < 0.001) (Figure 1). In addition, NA against the Delta variant was higher compared to the Omicron one all over time (SV, T0 and T1: *p* < 0.001; T2 *p* < 0.01; SIV, T0: *p* < 0.05; T1 and T2: *p* < 0.01) (Figure 1).

**Figure 2:**
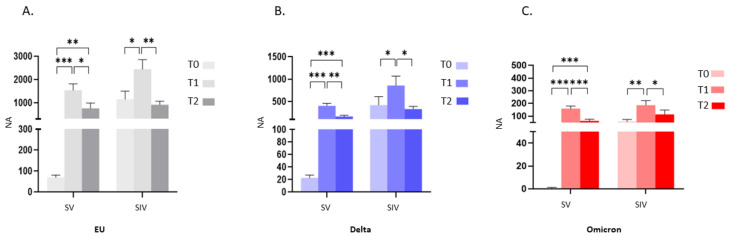
Plasma neutralizing activity (NA) trend over time. NA significantly increased from T0 to T1 and decreased from T1 to T2 against the EU (**A**), Delta (**B**) and Omicron (**C**) variants in both SV and SIV. Mean values ±SE are reported. * *p* < 0.05, ** *p* < 0.01, *** *p* < 0.001.

The analyses of NA over time revealed a significant increase from T0 to T1 against all tested viral strains in both SV (*p* < 0.001), and SIV (EU and Delta: *p* < 0.05; Omicron: *p* < 0.01) (Figure 2A–C), indicating that repeated exposure to the antigen, through either infection or booster vaccination, augments immune responses to SARS-CoV-2. Despite this, in just a 3-months period, the protective immunity at the systemic level considerably waned. Indeed, despite still being detectable in almost all the enrolled subjects, NA against SARS-CoV-2 EU strain and the two VOCs significantly declined from T1 to T2 in SV (EU: *p* < 0.05; Delta: *p* < 0.01; Omicron: *p* < 0.001) as well as SIV (EU: *p* < 0.01; Delta: *p* < 0.05; Omicron: *p* < 0.05) (Figure 2A–C).

**Figure 3:**
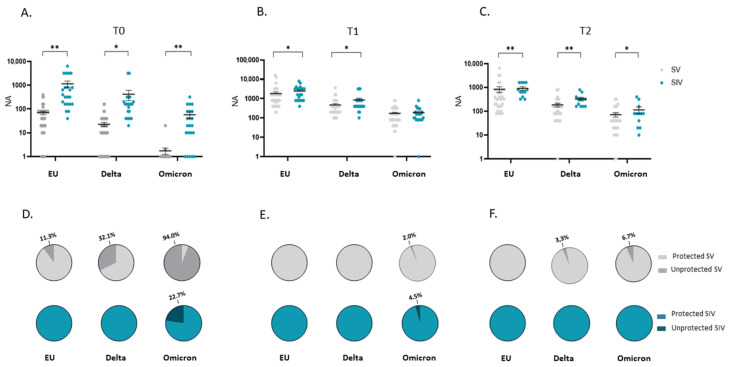
Comparison of plasma neutralizing activity (NA) in SARS-CoV-2 Vaccinated Subjects (SV) and SARS-CoV-2 Infected and Vaccinated Subjects (SIV). NA in plasma from SIV was higher than in SV against all the examined VOCs at all time points: T0 (**A**), T1 (**B**), and T2 (**C**). The pie charts show the percentage of SV (grey) and SIV (Blue) lacking serum NA against the EU, Delta and Omicron variants at T0 (**D**), T1 (**E**), and T2 (**F**), respectively. * *p* < 0.05, ** *p* < 0.01.

Notably, the mean NA value at T0 in plasma from SIV was higher compared with SV against the EU strain (16.6-fold; *p* < 0.01) as well as the Delta (18.5-fold; *p* < 0.05) and the Omicron (70.9-fold; *p* < 0.01) variants (Figure 3A). Even more relevant, the percentage of subjects who did not display NA against all SARS-CoV-2 strains in plasma before the third dose booster administration (T0) was consistently higher in SV (EU: 6/53 = 11.3%; Delta: 17/53 = 32.1%; Omicron: 49/52 = 94%) compared to SIV (EU:0/22 = 0%; Delta: 0/22 = 0%; Omicron: 5/22 = 22.7%) (Figure 3D). The higher responsiveness of SIV persisted even at T1 (EU: 1.6-fold, *p* < 0.05; Delta: 2.2-fold, *p* < 0.05) (Figure 3B) and T2 (EU: 1.2-fold, *p* < 0.01; Delta: 1.8-fold, *p* < 0.01; Omicron: 1.6-fold, *p* < 0.05) (Figure 3C). However, following booster dose administration, almost all subjects developed a NA above the detection limit; indeed, only a minority of both SV and SIV lacked NA at T1 (SV: EU: 0/50 = 0%; Delta: 0/50 = 0%; Omicron: 1/49 = 2%) (SIV: 0/22 = EU: 0%; Delta: 0/22 = 0%; Omicron: 1/21 = 4%) (Figure 3E) and T2 (SV: EU: 0/29 = 0%; Delta: 1/30 = 3.3%; Omicron: 2/30 = 6.7%) (SIV: EU: 0%; Delta: 0%; Omicron: 0%) (Figure 3F).

Overall, these data suggest that a booster vaccine dose triggers a protective NA against each of the tested SARS-CoV-2 strains in all the enrolled subjects, but its effectiveness seems more robust in subjects who were previously infected by the virus.

**Figure 4:**
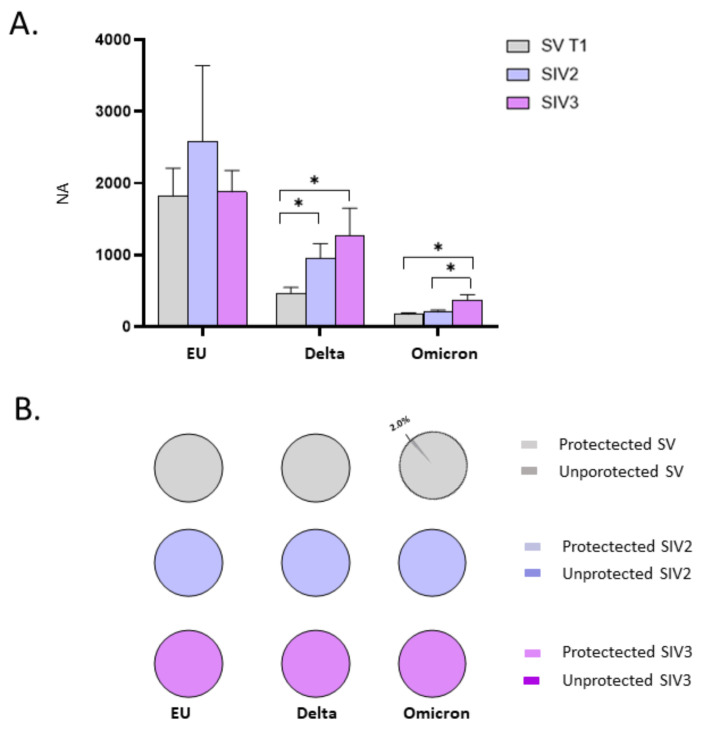
Comparison of neutralizing activity (NA) in plasma specimens from SARS-CoV-2 Infected after 2 (SIV2), or 3 doses of Vaccine (SIV3) and SARS-CoV-2 Vaccinated subjects (SV) at T1. NA in plasma from SIV was higher than in SV against all the examined VOCs (**A**). Pie charts show the percentage of SV (grey), SIV2 (light blue), and SIV3 (lilac) lacking NA against the EU, Delta, and Omicron variants, respectively (**B**). * *p* < 0.05.

Such an assumption is further strengthened by comparing NA in plasma specimens from SIV2, SIV3, and SV at T1. In particular, we observed a statistically significant increase in NA of SIV2 versus SV against the Delta variant (*p* < 0.05), and in SIV3 compared to SV against both the Delta and Omicron strains (*p* < 0.05) (Figure 4A). Besides, NA against the Omicron variant was significantly higher in SIV3 compared to SIV2 (*p* < 0.05) (Figure 4A). Conversely, no statistically significant differences were observed among SV, SIV2, and SIV3 NA against the EU strain, indicating that the booster dose is able to stimulate an immune response comparable to that prompted by natural infection against the original SARS-CoV-2 EU strain employed in the vaccine formulation, but not against the new emerging variants. Overall, 15 days post booster vaccine dose administration (SV at T1), or infection (SIV2, SIV3) all the subjects developed a NA, except for 1/49 SV, who lacked NA against the Omicron strain (NA negative SV: EU: 0/50 = 0%; Delta: 0/50 = 0%; Omicron: 1/49 = 4.5%; NA negative SIV2: EU: 0/7 = 0%; Delta: 0/7 = 0%; Omicron: 0/7 = 0%; NA negative SIV3: EU: 0/24 = 0%; Delta: 0/24 = 0%; Omicron: 0/24 = 0%) (Figure 4B).

The mean value of NA in the plasma specimens of the different enrolled groups over time are reported in Table 1.

### 2.2. SARS-CoV-2 NA in Saliva

NA in saliva from SV and SIV mirrored the trend registered in plasma specimens, though the NA titres were significantly lower (data not shown).

**Figure 5:**
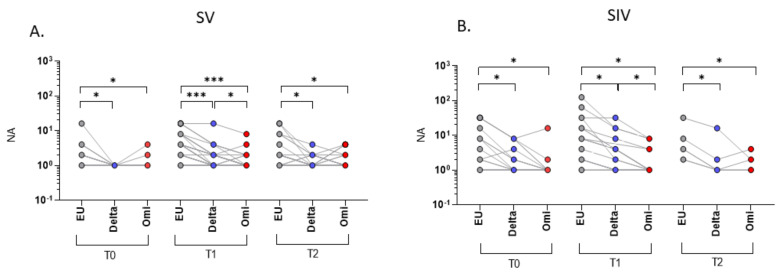
Saliva neutralizing activity (NA) correlations among different VOCs. NA correlation performed at different time points (T0, T1, and T2) showed higher NA against the EU variant compared to the Delta and Omicron (Omi) variants in plasma samples from both SARS-CoV-2 Vaccinated subjects (SV) (**A**) and SARS-CoV-2 Infected and Vaccinated subjects (SIV) (**B**). Lines connect the NA of each subject. * *p* < 0.05, *** *p* < 0.001.

NA in saliva was significantly higher against the EU strain compared to both Delta and Omicron variants in both SV (Delta: T0 and T2: *p* < 0.05; T1: *p* < 0.001) (Omicron: T0 and T2: *p* < 0.05; T1: *p* < 0.001) (Figure 5A) and SIV (*p* < 0.05 for all comparisons) (Figure 5B). Moreover, at T1, NA versus the Delta variant was significantly higher compared with the Omicron strain in both SV and SIV (*p* < 0.05) (Figure 5A,B).

**Figure 6:**
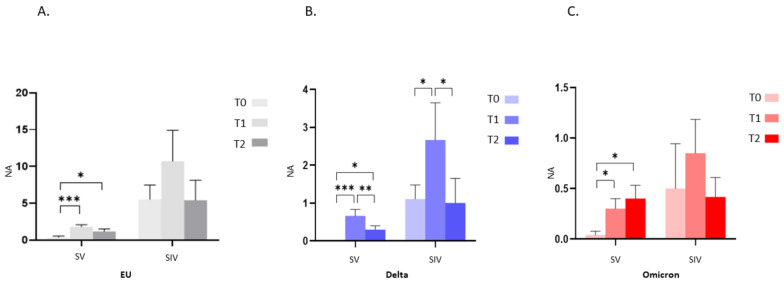
Saliva neutralizing activity (NA) trend over time. NA significantly increased from T0 to T1 and decreased from T1 to T2 against the EU (**A**), Delta (**B**) and Omicron (**C**) variants in both SV and SIV. Mean values ±SE are reported. * *p* < 0.05, ** *p* < 0.01, *** *p* < 0.001.

By studying the NA trend over time in saliva specimens, we observed that in SV, third dose vaccine administration increased mucosal NA from T0 to T1, reaching statistical significance against all: EU and Delta: *p* < 0.001; Omicron: *p* < 0.05 (Figure 6A–C). However, mirroring the scenario observed in plasma samples, protective NA dropped from T1 to T2; yet such a decrease was statistically significant only for the Delta variant (Delta: *p* < 0.01) (Figure 6A–C). Though the trend of oral NA over time was similar in SIV (an increase from T0 to T1 and a decrease from T1 to T2), no statistically significant differences were recorded at all-time points, except for the Delta variant (T0 vs. T1 and T1 vs. T2: *p* < 0.05) (Figure 6B).

**Figure 7:**
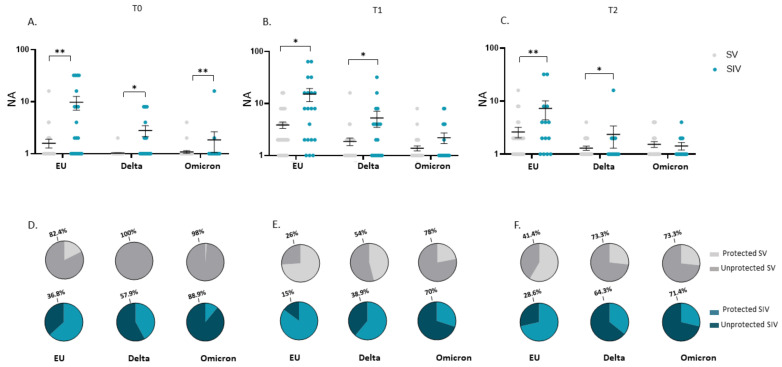
Comparison of saliva neutralizing activity (NA) in SARS-CoV-2 Vaccinated Subjects (SV) and SARS-CoV-2 Vaccinated and infected Subjects (SIV). NA in saliva from SIV was higher than in SV against all the examined VOCs at all time points: T0 (**A**), T1 (**B**), and T2 (**C**). Mean values ± SE are reported. * *p* < 0.05, ** *p* < 0.01. Pie charts showing the percentage of SV (grey) and SIV (blue) who do not have NA against the EU, Delta, and Omicron variants at T0 (**D**), T1 (**E**), and T2 (**F**). * *p* < 0.05, ** *p* < 0.01.

As in plasma samples, at T0, saliva from SV showed a reduced NA against all strains compared to SIV (EU: 14.8-fold, *p* < 0.01; Delta: 10-fold, *p* < 0.05; Omicron: 12.7, *p* < 0.01) (Figure 7A); also, at T0 the percentage of individuals lacking NA in the saliva was remarkably higher in SV (EU: 42/51 = 82.4%; Delta: 51/51 = 100%; Omicron: 50/51 = 98%) compared to SIV (EU:7/19 = 36.8%; Delta: 11/19 = 57.9%; Omicron: 16/18 = 88.9%) (Figure 7D). This very same difference persisted following boost dose administration at both T1 (NA of SIV vs. SV-EU: 5.9-fold, *p* < 0.05; Delta: 4-fold, *p* < 0.05) (Figure 7B) and T2 (NA of SIV vs. SV- EU: 3.9-fold, *p* < 0.01; Delta: 2.8-fold, *p* < 0.05) except for the Omicron variant (Figure 7C). Again, the percentage of individuals lacking NA in the oral mucosa against the SARS-CoV-2 EU strain and the two VOCs was higher in SV compared to SIV at both T1 (SV: EU: 13/50 = 26%; Delta: 27/50 = 54%; Omicron: 39/50 = 78%) (SIV: EU: 3/20 = 15%; Delta: 7/18 = 38%; Omicron: 14/20 = 70%) (Figure 7E) and T2 (SV: EU: 12/29 = 41%; Delta: 22/30 = 73.3%; Omicron: 22/30 = 73.3%) (SIV: EU: 4/14 = 28.6%; Delta: 9/14 = 64.3%; Omicron: 10/14 = 71.4%) (Figure 7F).

**Figure 8:**
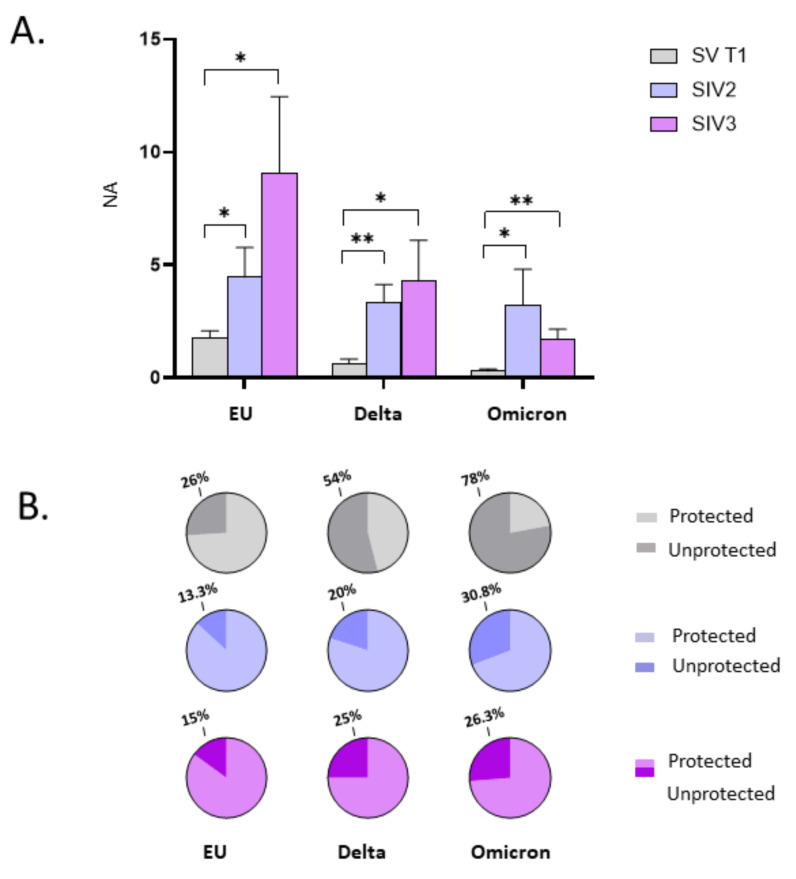
Comparison of neutralizing activity (NA) in saliva specimens from SARS-CoV-2 Infected after 2 (SIV2), or 3 doses of Vaccine (SIV3) and SARS-CoV-2 Vaccinated subjects (SV) at T1. NA against the EU, Delta, and Omicron variants in saliva samples from SIV2 and SIV3 was higher in comparison to SV samples at T1 (**A**). Pie charts show the percentage of SV (grey), SIV2 (light blue), and SIV3 (lilac) lacking salivary NA against the EU, Delta and Omicron variants, respectively (**B**). Mean values ± SE are reported. * *p* < 0.05, ** *p* < 0.01.

The observation that the booster dose prompts a more vigorous and long-lasting NA against SARS-CoV-2 in the oral mucosa of subjects who are naturally infected, was further confirmed by data comparing the NA in SIV2, SIV3, and SV at T1. Indeed, in SV at T1 oral NA against each of the tested SARS-CoV-2 strains was lower compared to SIV2 and SIV3 groups (EU, SV vs. both SIV2 and SIV3: *p* < 0.05; Delta, SV vs. SIV2: *p* < 0.001; SV vs. SIV3: *p* < 0.05; Omicron, EU vs. SIV2: *p* < 0.05; EU vs. SIV3: *p* < 0.001) (Figure 8A). Of note, the percentage of SV who did not develop NA in the oral mucosa at T1 (EU: 26%; Delta: 54%; Omicron: 58%) was nearly 2-fold compared to that registered in SIV2 (EU: 2/15 = 13.3%; Delta: 3/15 = 20%; Omicron: 4/15 = 30.8%) and SIV3 (EU: 3/20 = 15%; Delta: 5/20 = 25%; Omicron:5/19 = 26.3%), confirming that vaccine administration is less efficient in triggering protective immunity within the oral mucosa in naïve-to-infection subjects compared to subjects with prior SARS-CoV-2 infection (Figure 8B).

The mean value of NA in saliva samples of the different enrolled groups over time are reported in Table 2.

### 2.3. SARS-CoV-2 NA Correlation in Plasma and Saliva

**Figure 9:**
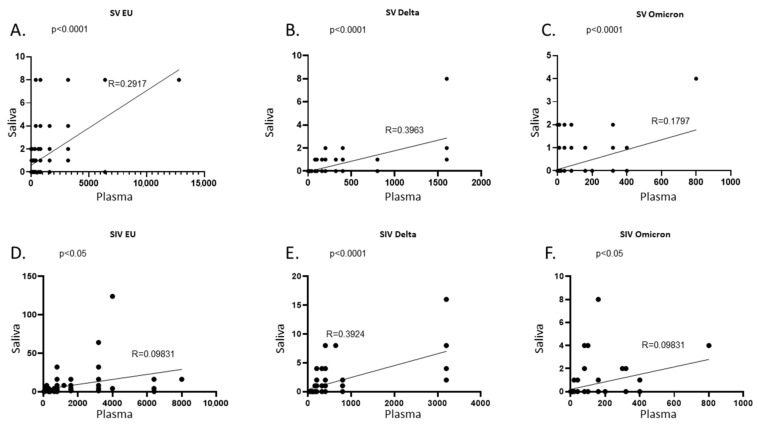
Correlation between NA in plasma and saliva samples. In SARS-CoV-2 Vaccinated subjects (SV) plasma and saliva NA against the EU (**A**), Delta (**B**), and Omicron (**C**) variants were positively correlated. Likewise, in SARS-CoV-2 Vaccinated and infected subjects (SIV) a positive correlation was found between plasma and saliva NA against the EU (**D**), Delta (**E**), and Omicron (**F**) variants. NA at all time points were considered in each graph.

By comparing the amount of NA over time in plasma and saliva of all the subjects enrolled in the study, we observed a positive correlation against the EU strain and the two VOCs in SV (*p* < 0.0001 for all) (Figure 9A–C) as well as SIV (EU and Omicron: *p* < 0.05; Delta: *p* < 0.001) (Figure 9D–F). However, although the quantification of SARS-CoV-2 NA in saliva seems to mirror the trend of NA in plasma, the correlation coefficient (R) does not suggest a strong agreement between the two variables. To deepen the correlation between saliva and plasma NA, further analyses on larger cohorts will be necessary.

No correlation with sex or age was detected with NA, neither in plasma nor in saliva samples from the enrolled groups (data not shown).

### 2.4. Cytokines and Chemokines in Plasma and Saliva

Cytokines and chemokines production was monitored by multiplex assay in both plasma and saliva samples at all-time points in all the enrolled subjects. No statistically significant differences were reported by comparing all the enrolled groups (data not shown). However, it is worth noting that, in saliva samples from SIV2 and SIV3, the production of proinflammatory cytokines (IL1-Ra, IL-6, IL-7, IL-17, GCS-F, IFN-γ, MCP-1, TNFα) was increased compared to samples from SV at T1. This suggests that despite having recovered from SARS-CoV-2 infection, immune activation persists in the oral mucosa (Supplementary Figure S1).

## 3. Discussion

COVID-19 vaccines provide strong protection against severe disease and death; however, SARS-CoV-2 specific immunity seems to progressively wane. Accordingly, several countries whose population has already been largely protected by the vaccine, have offered booster doses whose long-term protective effect is currently under investigation. In this perspective, the main purpose of the present study was to monitor the trend of SARS-CoV-2 neutralizing activity (NA) induced by a booster dose of BNT162b2 COVID-19 vaccine compared to natural infection. As the oral mucosa represents the primary site of virus acquisition but also the main defensive barrier to control viral spreading, such analyses were performed at both systemic and oral level.

As expected, in both plasma and saliva samples, NA significantly increases from T0 to T1 independently of a previous SARS-CoV-2 infection, indicating the effectiveness of a third dose administration, which deserves to be included in the standard vaccination schedule and not merely considered as an optional booster dose. Yet, after only three months, NA substantially declined in both plasma and oral cavity. Furthermore, our preliminary results of NA after 6-months booster dose administration suggest a subsequent and progressive drop of SARS-CoV-2 NA over time, in those subjects who were not infected in the meantime (data not shown). A remaining open matter is whether the residual NA induced by the third dose, which is still present in almost all the enrolled subjects at systemic level, will be able to protect from severe COVID-19 and/or infection, or whether a further booster vaccine dose administration is required to produce a long-term, defensive immune response. Furthermore, to get a 360° overview, this information should be mandatorily combined with data on SARS-CoV-2-specific T-Cell mediated immunity, whose long-term maintenance has already been documented in Ref. [22]. As new variants have been continuously emerging, assessing the role played by this residual immunity on these VOCs is mandatory as well. Indeed, our results show that BNT162b2 vaccine-induced NA is generally robust against the EU strain while it becomes weaker against the Delta and Omicron variants, in both serum and saliva. This is not surprising when considering the phenomenon of virus-induced immune evasion, which was recently documented for the Delta and Omicron variants compared to the SARS-CoV-2 EU strain, against which the original vaccine was developed [12]. Several studies already described the mutations responsible for evasion of the immune response. The Delta variant presents different mutations on the Spike protein such as T19R, G142D, D156e157, R158G, L452R, T478K, D614G, P681R, and D950 [23,24], while the Omicron variant carries 32 mutations on the Spike protein [25]. These changes may dramatically enhance VOC’s ability to evade the current vaccines that still employ the same original spike antigen for both initial rollouts and booster doses. The changing face of the novel VOCs may therefore represent a new challenge for scientists to modify existing vaccines to better tackle the antigenic drifts of SARS-CoV-2.

Further valuable information emerging from this study concerns the role played by natural infection in the preservation of a shielding SARS-CoV-2 specific immunity. First and foremost, at T0, nearly 6 months from the administration of the previous vaccine dose, the average NA level, as well as the percentage of people who still maintained a NA above the threshold at systemic level, is significantly higher in people who developed COVID-19 in addition to vaccination. Such a discrepancy is even more evident when considering the NA against the Delta and mostly the Omicron variant. Indeed, nearly all of the SV at T0 did not display NA against the latter. This substantiates the evidence that the immune response elicited by the current vaccine against strains other than the one used in the original formula, unlike natural infection, provides only moderate protection and/or it rapidly wanes [15]. Of note, SIV3 who were infected from the beginning of 2022, when the Omicron variant was predominant in Italy, showed stronger NA against this strain compared to SIV even after the administration of the booster dose (T1). This observation casts the hypothesis that the monitoring of NA at both oral and systemic level potentially allows to discriminate the variant responsible for the infection.

Although almost all subjects developed NA at the systemic level at T1, the significant gap in NA amount between SV and SIV was maintained at 15 days and increased at 3 months from vaccine administration. Likewise, we observed that NA developed by SIV2 and SIV3 at 15 days post recovery was significantly higher compared to NA in SV at 15 days post booster dose administration, substantiating the previously emphasized concept of a more robust and durable hybrid immunity, being triggered from both vaccination and infection, compared to that induced by either vaccination or infection alone [21,26,27,28]. The novelty of the present study consists of extending this evidence from systemic to the oral level, allowing us to draw some remarkable observations. First, at T0, SIV display a higher oral NA compared to SV against all strains, possibly as a consequence of a persistent exposure to virus antigens in the oral mucosa, possibly due to a sub-clinical infection as reported in the intestine of convalesced COVID-19 subjects up to 4 months after infection [29]. Based on this observation we would also speculate on the likelihood that an oral administration of the vaccine could evoke the same long-lasting mucosal responsiveness in vaccinated subjects, which in turn could increase resistance to new viral exposures [30,31]. In line with this observation, NA in saliva from SIV is far more boosted following third dose administration compared to SV against the SARS-CoV-2 EU strain as well as the two VOCs. Notwithstanding, we also have to point out that not all the SIV displayed NA at T0, while NA was present in nearly all SIV2 and SIV3. This suggests that, although infection primarily occurs in the oral mucosa, oral immunity triggered by natural infection may wane over time in some subjects as well.

Our results are in line with a recent study by Darwich et al., showing that the level of IgA, which display a higher SARS-CoV-2 NA compared to IgG [29], is lower in the saliva of vaccinated people compared to previously exposed subjects [32]. We also observed that the saliva NA against any of the SARS-CoV-2 tested strain, was much lower in subjects who were naïve to SARS-CoV-2 infection even after booster dose administration. Overall, these data suggest that a combination of vaccination and prior infection offers the most effective protection against SARS-CoV-2, an observation that should be taken into account when scheduling the next steps for vaccine administration. This finding is consistent with data on salivary cytokine secretion, which albeit not significant, show a trend of higher immune-activation in SIV2 and SIV3 subjects compared to SV at T1, presumably driven by the recent infection or remnants thereof.

In our previous paper we disclosed a positive correlation between plasma and saliva NA [21], which tendency seems to be maintained in the present study. However, at T1, almost all subjects displayed NA at the systemic level but not in the oral mucosa, likely due to a limited capacity of the intramuscularly administered vaccine to properly stimulate mucosal immunity [33], a pattern shared by the administration of other intramuscular vaccines such as varicella [34] and cytomegalovirus [35]. Thus, saliva NA does not exactly mirror the systemic scenario and does not represent an affordable substitute to assess vaccine-induced NA. This observation further emphasizes the opportunity to test an oral vaccine which, in theory, would trigger a more robust response at mucosal level as already observed for vaccine against other pathogens such as polio [36] and influenza virus [37].

In conclusion, as currently it is uncertain whether or not higher levels of NA versus lower levels correlate with either acquisition or progression of infection, tracking vaccine-induced NA may provide a valuable element to ascertain this question mark and thus, make focused and aware decisions about future vaccination program. However, the take home message rising from this study is 2-fold and mainly concerns: (i) the need to update the vaccine formulation so as to make it able to protect even against the new emerging variants and (ii) to possibly set up new oral route of vaccine administration, which could ideally provide stronger stimulation of mucosal immunity and greater protection against SARS-CoV-2 infection.

## 4. Materials and Methods

### 4.1. Study Design

A longitudinal, observational study was designed to evaluate the development of humoral immunity in four different subject categories: (i) SARS-CoV-2-Vaccinated (SV) subjects, who received three vaccine doses, (ii) SARS-CoV-2-Vaccinated subjects who were also infected before the first dose of vaccine administration (SIV), and subjects who contracted SARS-CoV-2 infection after either (iii) two-doses of vaccine administration (SIV2) or (iv) three-doses of vaccine administration (SIV3). In SV and SIV, saliva and blood sampling were performed at T0 (before booster dose administration), T1 (15 days from booster dose administration) and T2 (90 days from booster dose administration). Specimens from SIV2 and SIV3 were taken after recovery [mean time after first positive SARS-CoV-2 swab (days) ± SD: 13 ± 3.5]. The sampling timing is summarized in Table 2.

The primary end point of the study was to monitor the course of neutralizing activity (NA) triggered by the administration of a third (booster) vaccine dose over time. Secondary end points were: (i) to evaluate virus NA in plasma and saliva specimens against the main VOCs at the time of the study: Delta and Omicron; (ii) to compare NA induced by vaccination and natural infection over time; (iii) to verify if the trend of NA in the oral cavity reflects NA at the systemic level. The study design is summarized in the Graphical Abstract.

### 4.2. Viral Strains and Cell Lines

SARS-CoV-2, including the lineage B.1 (EU) (accession number: EPI_ISL_412973), assumed as the comparator virus, the Delta variant (lineage B.1.617.2) (accession number: EPI_ISL_1970729) and Omicron variant (lineage B.1.1.529) (accession number: EPI_ISL_6777160) were isolated from positive nasopharyngeal swabs. All the strains were characterized by means of whole genome sequencing and the sequences were submitted to GISAID. The virus was expanded in VeroE6 cells (ATCC^®^ VERO C1008, CRL-1586™) and viral titres were determined by Median Tissue Culture Infectious Dose (TCID50) endpoint dilution assay as previously described [38]. All the assays with SARS-CoV-2 virus were performed in a BSL3 facility.

### 4.3. Study Population and Sample Collection

Plasma and saliva samples were obtained on a voluntary basis from health volunteers and students at the Medical School of Medicine, University of Milan, Italy. Subjects were enrolled at the immune-biology laboratory, University of Milan, (Italy) and included: 55 SARS-CoV-2 SV [mean age (years) ± DS: 32.7 ± 15.1; range: 18–81; female: 69%], 27 SIV [mean age (years) ± SD: 47.9 ± 21.0; range: 18–85; female: 59.26%], 16 SIV2 [mean age (years) ± SD: 37.9 ± 18.8; range: 18–78; female: 43.75%], and 24 SIV3 [mean age (years) ± SD: 47.8 ± 20.7; range: 18–78; female: 54.17%]. SARS-CoV-2 infection was determined by SARS-CoV-2 molecular test of nasopharyngeal swabs. The prevalent circulating SARS-CoV-2 strains at the time of infection of SIV and SIV3 were the EU and Omicron variants, respectively. Instead, SIV2 were infected in a period in which both the Delta and Omicron strains were circulating.

All the enrolled subjects were administered the BNT162b2 (Comirnaty) anti-SARS-CoV-2 vaccines. At T0, all the enrolled subjects had received two doses according to the specific vaccination schedules (BNT162b2: dose II administered 21 days after dose I), except for SIV who were vaccinated within 6 months from SARS-CoV-2 infection recovery and received just a single vaccine dose. SIV2 were not administered the third booster dose, as they contracted SARS-CoV-2 infection prior to/in the proximity of third booster dose vaccine scheduling. At T0, time from previous vaccination and infection was 5.9 ± 1.4 months and 14.5 ± 6.2 months, respectively, and no statistically significant differences were registered by comparing SIV and SV. Cohort features are reported in Supplementary Table S1.

Smokers and subjects affected by inflammatory diseases or undergoing immunosuppressive therapies were excluded from the study. The timeline of vaccine administration for SV and SIV is reported in the graphical abstract.

Plasma was obtained by centrifugation of whole blood at 1500 g for 10 min and storage at −20 °C until use. Plasma samples were incubated at 56 °C for 30 min and analysed using iFlash SARS-CoV-2 IgG and IgM (C86095G–C86095M–Shenzhen YHLO Biotech Co, Shenzhen, China) to exclude a possible ongoing asymptomatic infection since the assay targets both nucleocapsid and spike proteins. Only the subjects included in the SIV, SIV2, and SIV3 groups resulted to have SARS-CoV-2 N plus S antigens (data not shown).

According to the previously validated protocol [21], all saliva samples were collected by spitting, in the morning between 9 and 10 am, and always at least 1 h after meal and after repeated mouth-washing with water. Saliva was incubated at 56 °C for 10 min and centrifuged at 6000 g for 10 min. Supernatants were stored at −80°C until use. Participants were asked not to eat or drink at least 1 h prior to sample collection.

Ethical clearance was obtained from the University of Milan Ethics Committee (number 14/22). Written informed consent was obtained after receiving information about use of their biological samples. The biological material was anonymized.

### 4.4. SARS-CoV-2 Virus Neutralization Assay (vNTA)

The detailed set up of Virus Neutralization assay (vNTA) in plasma and saliva samples from all the subjects enrolled in the study was previously described [21]. Wells were scored to evaluate the degree of CPE compared to the virus control. Blue staining of wells indicated the presence of NA. Neutralizing titer corresponds to the maximum dilution with the reduction of 90% of CPE. This information, as well as the results, were considered positive if higher or equal to 1:10 serum titre [39,40] or 1:1 for saliva specimens. Neutralization activity (NA) was tested against SARS-CoV-2 B.1 (EU) strain, Delta (lineage B.1.617.2) variant and Omicron (lineage B.1.1.529) variant at three different time points: T0, T1 and T2.

### 4.5. Cytokine/Chemokine Quantification in Plasma and Saliva Samples by Multiplex ELISA

The concentration of 27 cytokines/chemokines was assessed on plasma and saliva specimens collected at different time points, from a subgroup of randomly selected vaccinated subjects (SV: n = 5; SIV: n = 5; SIV2 = 5; SIV3 = 5) using magnetic bead-based immunoassays (Bio-Rad, Hercules, CA, USA), according to the manufacturer’s protocol via Bio-Plex 200 technology (Bio-Rad, Hercules, CA, USA). Some of the targets had values above the standard range, and an arbitrary value of 10 000 pg/ml was assigned, while values under the detection limit were set as 0 pg/ml.

### 4.6. Statistical Analyses

For the study variables, medians and ranges were reported for quantitative variables, and absolute and relative frequencies were reported for categorical variables. The Student’s T-test and analysis of variance (ANOVA) were applied when appropriate for statistical analyses to compare variables among the analysed groups. Quantitative variables association was evaluated through Pearson correlation coefficient r. A *p*-value < 0.05 was set as cut-off for significance. The analyses were performed using GraphPad Prism 9.

All the procedures were carried out in accordance with the GLP guidelines adopted in our laboratories.

## Figures and Tables

**Table 1:** Mean value of the neutralizing activity (NA) in plasma and saliva samples of the different enrolled groups over time.

	T0						T1						T2					
	Plasma NA Mean ± SE			Saliva NA Mean ± SE			Plasma NA Mean ± SE			Saliva NA Mean ± SE			Plasma NA Mean ± SE			Saliva NA Mean ± SE		
	EU	Delta	Omicron	EU	Delta	Omicron	EU	Delta	Omicron	EU	Delta	Omicron	EU	Delta	Omicron	EU	Delta	Omicron
SV	69 ± 11.5	23 ± 4.8	0.8 ± 0.6	0.4 ± 0.2	0.0 ± 0	0.0 ± 0	1533 ± 257.8	404 ± 52.0	160 ± 20.4	1.8 ± 0.3	0.7 ± 0.2	0.3 ± 0.1	756 ± 210.5	164 ± 28.2	63 ± 13.0	1.2 ± 0.3	0.3 ± 0.1	0.4 ± 0.1
SIV	1150 ± 341.2	419 ± 192.9	57.3 ± 16.9	5.5 ± 1.9	1.1 ± 0.4	0.5 ± 0.4	2436 ± 411.0	859 ± 208.1	187 ± 36.0	7.9 ± 3.2	2.7 ± 0.9	0.9 ± 0.3	892 ± 137.4	300 ± 54.1	99 ± 31.9	4.6 ± 2.4	0.9 ± 0.6	0.4 ± 0.2
SIV2							2583 ± 1019.8	954 ± 204.3	204 ± 29.4	4 ± 1.3	3 ± 0.8	3 ± 1.6						
SIV3							1950 ± 263.0	1455 ± 512.5	431 ± 100.5	11 ± 4.4	5 ± 2.3	2 ± 0.5						

**Table 2:** Meaning of the acronyms of the enrolled groups and the sampling time.

		Sampling Time		
Symbol	Description	T0	T1	T2
SV	Uninfected, SARS-CoV-2-booster dose administration	Before booster dose administration	15 days post booster dose administration	90 days post booster dose administration
SIV	Infected prior to SARS-CoV-2 booster dose administration	Before booster dose administration	15 days post booster dose administration	90 days post booster dose administration
SIV2	Infected after the 2nd SARS-CoV-2 vaccine dose administration		15 days post-infection	
SIV3	Infected post SARS-CoV-2 booster dose administration		15 days post-infection	

## Data Availability

Data that support the findings of the present study are available upon reasonable request to the corresponding author.

## Supplementary Materials

The following supporting information can be downloaded at: https://www.mdpi.com/article/10.3390/ijms232214341/s1, Figure S1. Cytokine secretion in saliva samples from SARS-CoV-2 Vaccinated Subjects (SV) at T0, SARS-CoV-2 infected Subjects after 2 doses (SIV2), or 3 doses of vaccine (SIV3); Table S1. Cohort study features.

## Abbreviations

ACE2	Angiotensin-converting enzyme 2
NA	Neutralizing Activity
SV	SARS-CoV-2 vaccinated
SIV	SARS-CoV-2 infected and vaccinated
SIV2	SARS-CoV-2 vaccinated and infected after 2 doses of vaccine
SIV3	SARS-CoV-2 vaccinated and infected after 2 doses of vaccine
vNTA	Virus Neutralization assay
VOCs	Variants of concern

## References

[1] Hamming I., Timens W., Bulthuis M.L.C., Lely A.T., Navis G.J., van Goor H. (2004). Tissue Distribution of ACE2 Protein, the Functional Receptor for SARS Coronavirus. A First Step in Understanding SARS Pathogenesis. J. Pathol..

[2] Xu H., Zhong L., Deng J., Peng J., Dan H., Zeng X., Li T., Chen Q. (2020). High Expression of ACE2 Receptor of 2019-NCoV on the Epithelial Cells of Oral Mucosa. Int. J. Oral Sci..

[3] Challenger J.D. (2022). Modelling Upper Respiratory Viral Load Dynamics of SARS-CoV-2. BMC Med..

[4] Ligtenberg A.J.M., Veerman E.C.I., Nieuw Amerongen A.V. (2000). A Role for Lewis a Antigens on Salivary Agglutinin in Binding to Streptococcus Mutans. Antonie Leeuwenhoek.

[5] Presland R.B., Jurevic R.J. (2002). Making Sense of the Epithelial Barrier: What Molecular Biology and Genetics Tell Us about the Functions of Oral Mucosal and Epidermal Tissues. J. Dent. Educ..

[6] Amerongen A.N., Veerman E. (2002). Saliva the Defender of the Oral Cavity. Oral Dis..

[7] Abiko Y., Saitoh M., Nishimura M., Yamazaki M., Sawamura D., Kaku T. (2007). Role of Beta-Defensins in Oral Epithelial Health and Disease. Med. Mol. Morphol..

[8] Akira S. (2009). Pathogen Recognition by Innate Immunity and Its Signaling. Proc. Jpn. Acad. Ser. B Phys. Biol. Sci..

[9] Chung H., He S., Nasreen S., Sundaram M.E., Buchan S.A., Wilson S.E., Chen B., Calzavara A., Fell D.B., Austin P.C. (2021). Effectiveness of BNT162b2 and MRNA-1273 COVID-19 Vaccines against Symptomatic SARS-CoV-2 Infection and Severe COVID-19 Outcomes in Ontario, Canada: Test Negative Design Study. BMJ.

[10] Dagan N., Barda N., Kepten E., Miron O., Perchik S., Katz M.A., Hernán M.A., Lipsitch M., Reis B., Balicer R.D. (2021). BNT162b2 MRNA COVID-19 Vaccine in a Nationwide Mass Vaccination Setting. N. Engl. J. Med..

[11] Khoury D.S., Cromer D., Reynaldi A., Schlub T.E., Wheatley A.K., Juno J.A., Subbarao K., Kent S.J., Triccas J.A., Davenport M.P. (2021). Neutralizing Antibody Levels Are Highly Predictive of Immune Protection from Symptomatic SARS-CoV-2 Infection. Nat. Med..

[12] Mileto D., Fenizia C., Cutrera M., Gagliardi G., Gigantiello A., De Silvestri A., Rizzo A., Mancon A., Bianchi M., De Poli F. (2021). SARS-CoV-2 MRNA Vaccine BNT162b2 Triggers a Consistent Cross-Variant Humoral and Cellular Response. Emerg. Microbes Infect..

[13] Björk J., Inghammar M., Moghaddassi M., Rasmussen M., Malmqvist U., Kahn F. (2022). High Level of Protection against COVID-19 after Two Doses of BNT162b2 Vaccine in the Working Age Population—First Results from a Cohort Study in Southern Sweden. Infect. Dis..

[14] Hall V., Foulkes S., Insalata F., Kirwan P., Saei A., Atti A., Wellington E., Khawam J., Munro K., Cole M. (2022). Protection against SARS-CoV-2 after COVID-19 Vaccination and Previous Infection. N. Engl. J. Med..

[15] Mileto D., Micheli V., Fenizia C., Cutrera M., Gagliardi G., Mancon A., Bracchitta F., De Silvestri A., Rizzardini G., Lombardi A. (2022). Reduced Neutralization of SARS-CoV-2 Omicron Variant by BNT162b2 Vaccinees’ Sera: A Preliminary Evaluation. Emerg. Microbes Infect..

[16] Wu K., Werner A.P., Moliva J.I., Koch M., Choi A., Stewart-Jones G.B.E., Bennett H., Boyoglu-Barnum S., Shi W., Graham B.S. (2021). MRNA-1273 Vaccine Induces Neutralizing Antibodies against Spike Mutants from Global SARS-CoV-2 Variants. BioRxiv.

[17] Weisblum Y., Schmidt F., Zhang F., DaSilva J., Poston D., Lorenzi J.C., Muecksch F., Rutkowska M., Hoffmann H.-H., Michailidis E. (2020). Escape from Neutralizing Antibodies by SARS-CoV-2 Spike Protein Variants. eLife.

[18] Wibmer C.K., Ayres F., Hermanus T., Madzivhandila M., Kgagudi P., Oosthuysen B., Lambson B.E., de Oliveira T., Vermeulen M., van der Berg K. (2021). SARS-CoV-2 501Y.V2 Escapes Neutralization by South African COVID-19 Donor Plasma. Nat. Med..

[19] Voysey M., Clemens S.A.C., Madhi S.A., Weckx L.Y., Folegatti P.M., Aley P.K., Angus B., Baillie V.L., Barnabas S.L., Bhorat Q.E. (2021). Single-Dose Administration and the Influence of the Timing of the Booster Dose on Immunogenicity and Efficacy of ChAdOx1 NCoV-19 (AZD1222) Vaccine: A Pooled Analysis of Four Randomised Trials. Lancet.

[20] Garcia-Beltran W.F., Lam E.C., Denis K., Nitido A.D., Garcia Z.H., Hauser B.M., Feldman J., Pavlovic M.N., Gregory D.J., Poznansky M.C. (2021). Multiple SARS-CoV-2 Variants Escape Neutralization by Vaccine-Induced Humoral Immunity. Cell.

[21] Garziano M., Utyro O., Poliseno M., Santantonio T.A., Saulle I., Strizzi S., Lo Caputo S., Clerici M., Introini A., Biasin M. (2022). Natural SARS-CoV-2 Infection Affects Neutralizing Activity in Saliva of Vaccinees. Front. Immunol..

[22] Moss P. (2022). The T Cell Immune Response against SARS-CoV-2. Nat. Immunol..

[23] Baral P., Bhattarai N., Hossen M.L., Stebliankin V., Gerstman B.S., Narasimhan G., Chapagain P.P. (2021). Mutation-Induced Changes in the Receptor-Binding Interface of the SARS-CoV-2 Delta Variant B.1.617.2 and Implications for Immune Evasion. Biochem. Biophys. Res. Commun..

[24] Yang J., Hu X., Wang W., Yang Y., Zhang X., Fang W., Zhang L., Li S., Gu B. (2022). RT-LAMP Assay for Rapid Detection of the R203M Mutation in SARS-CoV-2 Delta Variant. Emerg. Microbes Infect..

[25] Chen J., Wang R., Gilby N.B., Wei G.-W. (2021). Omicron (B.1.1.529): Infectivity, Vaccine Breakthrough, and Antibody Resistance. arXiv.

[26] Nordström P., Ballin M., Nordström A. (2022). Risk of SARS-CoV-2 Reinfection and COVID-19 Hospitalisation in Individuals with Natural and Hybrid Immunity: A Retrospective, Total Population Cohort Study in Sweden. Lancet Infect. Dis..

[27] Shenoy P., Ahmed S., Paul A., Cherian S., Umesh R., Shenoy V., Vijayan A., Babu S., Nivin S., Thambi A. (2022). Hybrid Immunity versus Vaccine-Induced Immunity against SARS-CoV-2 in Patients with Autoimmune Rheumatic Diseases. Lancet Rheumatol..

[28] Goldberg Y., Mandel M., Bar-On Y.M., Bodenheimer O., Freedman L.S., Ash N., Alroy-Preis S., Huppert A., Milo R. (2022). Protection and Waning of Natural and Hybrid Immunity to SARS-CoV-2. N. Engl. J. Med..

[29] Sterlin D., Mathian A., Miyara M., Mohr A., Anna F., Claër L., Quentric P., Fadlallah J., Devilliers H., Ghillani P. (2021). IgA Dominates the Early Neutralizing Antibody Response to SARS-CoV-2. Sci. Transl. Med..

[30] Focosi D., Maggi F., Casadevall A. (2022). Mucosal Vaccines, Sterilizing Immunity, and the Future of SARS-CoV-2 Virulence. Viruses.

[31] Russell M.W., Mestecky J. (2022). Mucosal Immunity: The Missing Link in Comprehending SARS-CoV-2 Infection and Transmission. Front. Immunol..

[32] Darwich A., Pozzi C., Fornasa G., Lizier M., Azzolini E., Spadoni I., Carli F., Voza A., Desai A., Ferrero C. (2022). BNT162b2 Vaccine Induces Antibody Release in Saliva: A Possible Role for Mucosal Viral Protection?. EMBO Mol. Med..

[33] Su F., Patel G.B., Hu S., Chen W. (2016). Induction of Mucosal Immunity through Systemic Immunization: Phantom or Reality?. Hum. Vaccines Immunother..

[34] Terada K., Niizuma T., Yagi Y., Miyashima H., Kataoka N., Sadahiro T. (2000). Low Induction of Varicella-Zoster Virus-Specific Secretory IgA Antibody after Vaccination. J. Med. Virol..

[35] Saccoccio F.M., Gallagher M.K., Adler S.P., McVoy M.A. (2011). Neutralizing Activity of Saliva against Cytomegalovirus. Clin. Vaccine Immunol..

[36] Baicus A. (2012). History of Polio Vaccination. World J. Virol..

[37] Tomar J., Patil H.P., Bracho G., Tonnis W.F., Frijlink H.W., Petrovsky N., Vanbever R., Huckriede A., Hinrichs W.L.J. (2018). Advax Augments B and T Cell Responses upon Influenza Vaccination via the Respiratory Tract and Enables Complete Protection of Mice against Lethal Influenza Virus Challenge. J. Control. Release.

[38] Barrow K.A., Rich L.M., Vanderwall E.R., Reeves S.R., Rathe J.A., White M.P., Debley J.S. (2021). Inactivation of Material from SARS-CoV-2-Infected Primary Airway Epithelial Cell Cultures. Methods Protoc..

[39] Lau E.H.Y., Tsang O.T.Y., Hui D.S.C., Kwan M.Y.W., Chan W., Chiu S.S., Ko R.L.W., Chan K.H., Cheng S.M.S., Perera R.A.P.M. (2021). Neutralizing Antibody Titres in SARS-CoV-2 Infections. Nat. Commun.

[40] Kohmer N., Westhaus S., Rühl C., Ciesek S., Rabenau H.F. (2020). Brief Clinical Evaluation of Six High-Throughput SARS-CoV-2 IgG Antibody Assays. J. Clin. Virol..

