# Supplementary material for: Saliva and Plasma Neutralizing Activity Induced by the Administration of a Third bnt162b2 Vaccine Dose
Source: Int J Mol Sci. 2022 Nov 18;23(22):14341. doi: 10.3390/ijms232214341 (PMC9693379; doi:10.3390/ijms232214341)
Supplement: Supplementary file 1 [file ijms-23-14341-s001.zip › ijms-2021991-supplementary.pdf]

Supplementary Table S1. Cohort study features.

	Subject N°	Gender	Age (years)	Time from Infection (months)	Time from 2nd dose (months)
SV	1	F	25	-	8
	2	F	28	-	9
	3	F	49	-	8
	4	M	58	-	8
	5	M	26	-	8
	6	F	25	-	8
	7	F	25	-	8
	8	M	25	-	8
	9	M	26	-	8
	10	M	26	-	8
	11	F	26	-	8
	12	F	26	-	7
	13	F	26	-	8
	14	F	20	-	7
	15	F	26	-	9
	16	F	20	-	5
	17	M	22	-	8
	18	F	23	-	8
	19	F	25	-	8
	20	M	28	-	8
	21	M	23	-	8
	22	F	24	-	8
	23	F	25	-	8
	24	F	51	-	6
	25	F	40	-	6
	26	F	68	-	6
	27	M	23	-	8
	28	F	34	-	8
	29	F	35	-	6
	30	M	40	-	6
	31	M	30	-	6
	32	F	33	-	6
	33	F	36	-	6
	34	F	20	-	5
	35	F	23	-	8
	36	F	24	-	8
	37	M	20	-	10
	38	F	23	-	9
	39	F	47	-	5
	40	M	22	-	6
	41	F	57	-	6
	42	M	40	-	6
	43	F	23	-	9

	44	F	26	-	8
	45	F	43	-	5
	46	F	37	-	6
	47	F	19	-	6
	48	F	81	-	7
	49	F	78	-	5
	50	M	47	-	5
	51	F	20	-	7
	52	F	35	-	5
	53	F	63	-	6
	54	M	31	-	6
	55	F	23	-	6
SIV	1	M	47	12	6
	2	F	60	13	6
	3	M	72	13	6
	4	F	55	12	6
	5	F	24	12	6
	6	M	25	12	5
	7	F	62	12	8
	8	M	71	12	7
	9	M	74	11	6
	10	M	40	11	5
	11	F	36	21	8
	12	F	18	24	6
	13	F	18	24	6
	14	F	50	24	10
	15	F	55	24	6
	16	F	71	11	4
	17	M	54	24	5
	18	M	18	24	5
	19	F	56	10	6
	20	F	81	10	5
	21	F	54	13	5
	22	M	85	10	5
	23	F	20	13	3
	24	M	20	11	5
	25	F	53	11	7
	26	F	25	17	5
	27	M	49	9	6
SIV2	1	F	25	0.6	9
	2	F	22	0.17	9
	3	F	34	0.43	8
	4	F	58	0.23	6
	5	M	21	0.33	5
	6	M	36	0.5	6
	7	F	34	1	7
	8	M	74	0.23	7
	9	M	78	0.5	6
	10	M	54	1	4
	11	F	43	0.7	5
	12	M	18	0.5	6

	13	M	36	0.7	4
	14	F	22	0.6	6
	15	M	29	0.53	6
	16	M	23	0.33	6
SIV3	1	F	51	0.5	6
	2	M	36	0.23	5
	3	F	31	0.5	8
	4	F	78	0.5	7
	5	F	50	0.43	6
	6	F	73	0.33	10
	7	M	76	0.6	7
	8	M	50	0.5	11
	9	M	66	0.7	10
	10	M	28	0.33	10
	11	F	32	0.27	9
	12	M	40	0.4	12
	13	M	18	0.57	5
	14	F	26	1	11
	15	F	26	1	14
	16	M	58	0.5	13
	17	M	27	0.7	9
	18	F	43	0.23	11
	19	F	78	0.3	11
	20	M	86	0.37	11
	21	M	30	0.27	12
	22	F	36	0.4	12
	23	F	23	0.5	8
	24	F	35	1	7

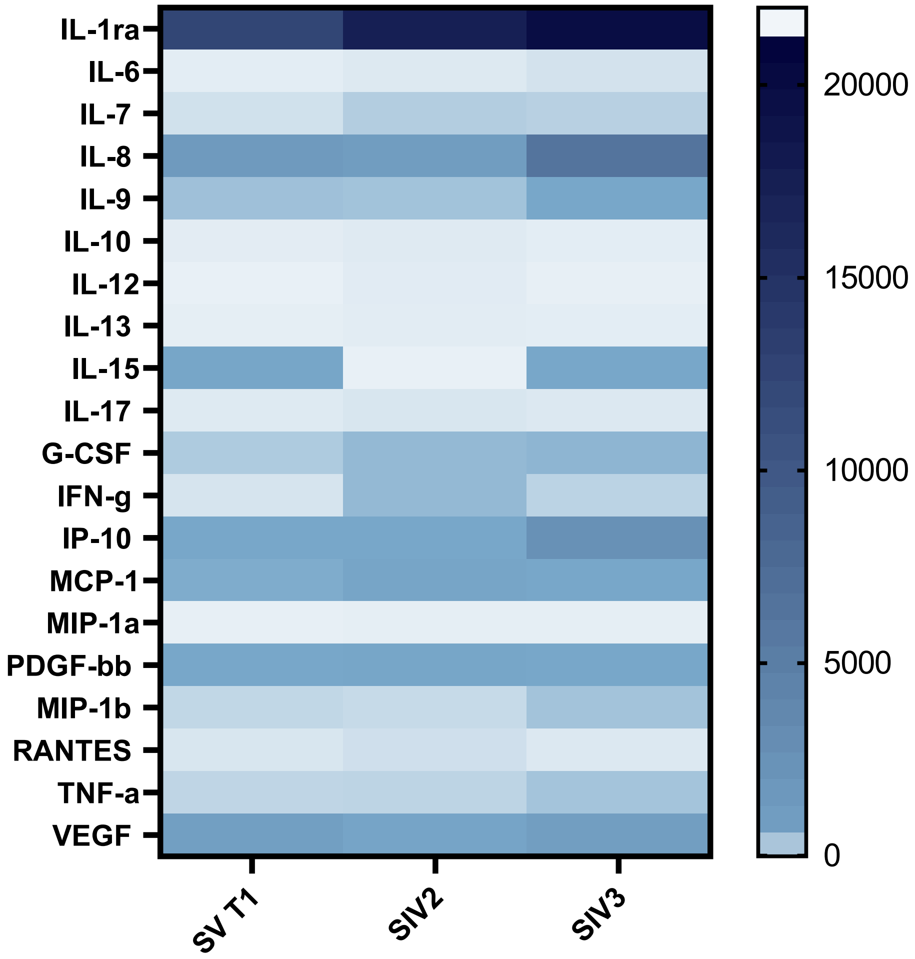

Supplementary Figure S1. Cytokine secretion in saliva samples from SARS-CoV-2 Vaccinated Subjects (SV) at T1, SARS-CoV-2 infected Subjects after 2 doses (SIV2), or 3 doses of vaccine (SIV3). Cytokine release in saliva samples from SIV2 and SIV3 was higher compared to vaccinated subjects at T1 suggesting the manteinance of a slight state of immune activation despite recent recovery
